# Pilot study of archimedes virtual bronchoscopic navigation system-guided biopsy to diagnose lung nodules in children

**DOI:** 10.3389/fped.2022.1053289

**Published:** 2023-02-02

**Authors:** Haiming Yang, Elijan Turgon, Yuena Pan, Xiaohui Wen, Xiaoyan Zhang, Yuelin Shen, Feng Wang

**Affiliations:** ^1^ Department No. 2 of Respiratory Diseases, Beijing Children's Hospital, Capital Medical University, National Center for Children's Health, Beijing, China; ^2^ Department of Respiratory Diseases, Children's Hospital of Xinjiang Uygur Autonomous Region, Xinjiang Hospital of Beijing Children's Hospita, Urumqi, China; ^3^ Department of Interventional Pulmonology, Beijing Children's Hospital, Capital Medical University, National Center for Children' Health, Beijing, China; ^4^ Department of Respiratory and Critical Care Medicine, Beijing Chaoyang Hospital, Capital Medical University, Beijing Institute of Respiratory Disease, Beijing, China

**Keywords:** bronchoscopic lung biopsy, children, archimedes, peripheral pulmonary lesions, new technique

## Abstract

**Background:**

Peripheral pulmonary lesions are uncommon in children. Bronchoscopy is a minimal invasive method to obtain a diagnostic lung biopsy. However, due to the lack of effective guidance methods, the diagnostic efficacy of transbronchial lung biopsy for peripheral solitary pulmonary diseases is still limited.

**Research question:**

Is the Archimedes virtual bronchoscopic navigation system safe and effective for the diagnosis of peripheral pulmonary lesions in children?

**Study design and methods:**

This pilot study retrospectively analyzed the clinical features, radiological characteristics, operation processes, intra-and postoperative complications, and pathological results of five children who underwent Archimedes-guided biopsy of peripheral pulmonary lesions in Beijing Children's Hospital from May 2021 to May 2022.

**Results:**

The cohort comprised five children (all males) with age of 7.1–15.8 years. A guide sheath was inserted through the bronchoscope under the guidance of Archimedes combined with radial endobronchial ultrasound to complete the biopsy under general anesthesia. The fused fluoroscopy technique was used to reconfirm the location of the forceps prior to biopsy in all children. The forceps reached the lesion under the guidance of the navigation and the samples were collected successfully in all children. Pathological examination of the biopsy specimens showed Epstein-Barr virus infection-related lymphoproliferative disease in one child, pulmonary metastasis of rhabdomyosarcoma in one child, and pulmonary vasculitis in one child; high-throughput sequencing of the biopsy tissue sample identified *Mycobacterium tuberculosis* (sequence no. 80) in one child and *Aspergillus* (sequence no. 40) in another child. All five children tolerated the biopsy procedure without developing postoperative complications, such as pneumothorax and hemoptysis.

**Interpretation:**

Archimedes-guided bronchoscopic lung biopsy is a feasible and efficient way to diagnose peripheral pulmonary lesions in children with manageable complications.

## Introduction

Pathological biopsy is currently the gold standard for the diagnosis of peripheral pulmonary lesions in clinical practice (1), but it is hard to obtain pathological samples rapidly and accurately from pediatric patients. Computed tomography (CT)-guided or ultrasound-guided percutaneous lung biopsy has a high diagnostic rate for peripheral pulmonary lesions, but often leads to complications. Similar to the field of adult pulmonology, the most common complications of percutaneous lung biopsy include pneumothorax, with reported rates up to 62%, and hemorrhage, with reported rates up to 56% (1, 2).

In addition, it is difficult to perform percutaneous lung biopsy when the lesions are hidden by the bony structures of the thoracic walls or are located adjacent to important organs. Another diagnostic method currently widely applied in clinical practice is transbronchial lung biopsy, and the newer cryo techniques for peripheral biopsies, which reportedly causes fewer complications than other methods (3). Based on our study, for adult patients, the most common complications of transbronchial lung biopsy are pneumothorax and bleeding with the incidence of 3.3% and 0.9%, respectively (4). However, compared with adults, it is more difficult to accurately approach the lesions in children with thinner bronchi; this leads to a low diagnostic rate for pediatric peripheral pulmonary lesions and has become a major diagnostic challenge in clinical practice (5). One study reported that the diagnostic rate of conventional bronchoscopy is only 34% in lesions with a diameter of less than 2 cm^4^. On chest CT images, peripheral pulmonary lesions appear as focal, quasi-circular, hyperdense opacities that can be single or multiple, generally without concomitant atelectasis, enlargement of hilar lymph nodes, or pleural effusion. Peripheral pulmonary lesions in children are caused by cancers, infection, autoimmune disorders, blood system diseases, vasculitis, and Crohn's disease, and are difficult to diagnose based on imaging alone in clinical practice, making biopsy the most important diagnostic method (6, 7). The methods used to biopsy peripheral pulmonary lesions in children include percutaneous lung biopsy, thoracoscopic lung biopsy, and bronchoscopic lung biopsy. Among these methods, bronchoscopic biopsy causes the least complications, but conventional bronchoscopy has an extremely low diagnostic rate for peripheral pulmonary lesions (2, 3). Due to the lack of effective guidance methods, the diagnostic efficacy of transbronchial lung biopsy for solitary peripheral pulmonary diseases is still limited. Furthermore, the airway subsegments are narrower in children than in adults, and lesions induced by different causes vary in blood volume and distribution of surrounding vessels. It is very challenging to select a reasonable biopsy site to reduce the risks of intraoperative hemorrhage and pneumothorax while collecting adequate diagnostic samples.

Therefore, it is vital to develop an effective diagnostic method with less fewer complications while accurately collecting adequate samples from children.

With the development of imaging techniques in recent years, the Archimedes Virtual Bronchoscopic Navigation (VBN) System has started to be used in the diagnosis of peripheral pulmonary lesions (8). Archimedes is a software system that reconstructs CT data into a three-dimensional (3D) model of the bronchial airways, vascular structures, and lungs, and provides virtual bronchial paths to guide the bronchoscope to the target lesions. The combination of Archimedes with radial endobronchial ultrasound (rEBUS) and fused fluoroscopy substantially increases the success rate of biopsy in the diagnosis of peripheral lung nodules in adults (9), but its application in children has not been reported. Therefore, the present study aimed to evaluate the application, feasibility, and safety of Archimedes-assisted lung biopsy in children.

## Study design and methods

### Patients

This retrospective case series included five children with solitary peripheral pulmonary lesions detected on CT that could not be definitely diagnosed by routine examination and conventional bronchoscopy who underwent Archimedes-guided bronchoscopic lung biopsy in the pneumology department of our hospital from May 2021 to May 2022. The Ethics Committee of Beijing Children's Hospital approved this study and waived the need for informed consent.

### Methods

#### Collection of clinical data

Data on general characteristics, clinical diagnoses, pulmonary images, endoscopic images, pathological results, and intra- and postoperative complications were collected from the Electronic Case System of the hospital.

#### Devices

The procedure was performed using a flexible bronchoscope (4.0 mm outer diameter, 2.0 mm working channel diameter, BF-P290; 2.8 mm outer diameter, 1.2 mm working channel diameter, BF-XP290. Olympus, Tokyo, Japan); Archimedes VBN System (Broncus, CA, United States); ultrasound processor (EU-ME2PP, Olympus, Tokyo, Japan), probe driving unit (MAJ-935, Olympus, Tokyo, Japan), rEBUS probe (UM-S20-17S, 20 MHz, outer diameter 1.7 mm; Olympus, Tokyo, Japan); guide sheath (outer diameter 1.95 mm, K-201; Olympus Tokyo, Japan); C-arm x-ray machine.

#### Procedures

**Figure 1:**
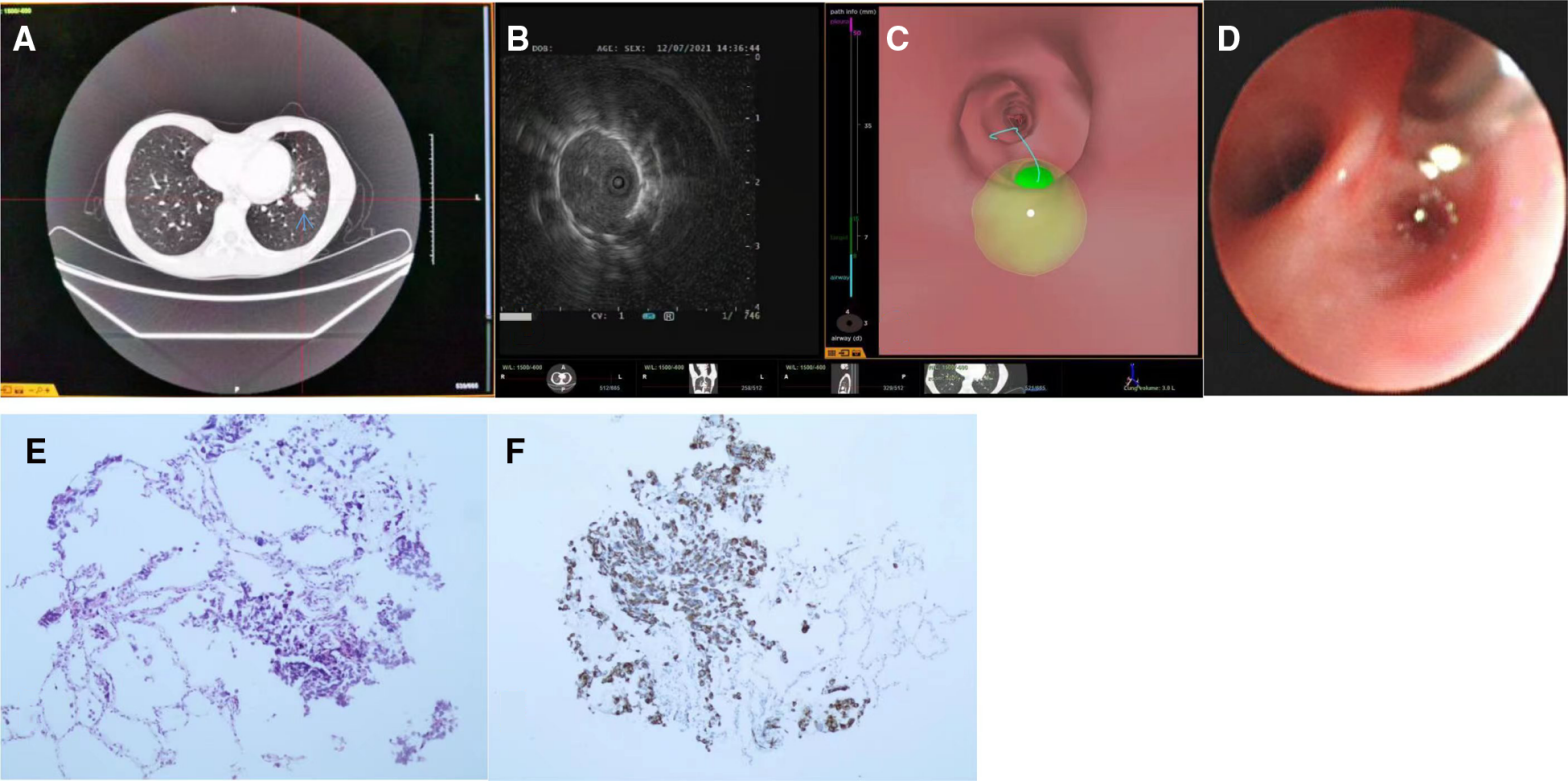
The operation process of archimedes virtual bronchoscopic navigation (VBN)-guided lung biopsy. (**A**) CT data requirements; (**B**) Lesions selected and marked (green region); (**C**) Selected navigation path; (**D**) Real-time guidance during navigation; (**E**) Reach the targeted location; (**F**) Radial endobronchial ultrasound image of the lesion.

High-resolution CT data (slice thickness 1 mm; interval space 0.6 mm; CT data requirements were shown in the Figure 1A) were inputted into the navigation system before surgery to reconstruct 3D bronchial and vascular models. When the lesions were marked (Figure 1B), the paths to the lesions were automatically calculated (Figure 1C). Before the bronchoscopic procedure, the operator could check and adjust the navigation path automatically formed by Archimedes' VBN system through CT scan image analysis and hand drawn navigation path. After the biopsy sites were selected, the relationships between the lesions and the surrounding vessels were assessed and the distances from the lesions to the pleura were measured. The procedures were completed with the child in the supine position under general anesthesia with laryngeal mask ventilation. The electronic guide paths and airway data were overlaid onto real-time endoscopic images to guide the endoscope to the target lesion to assist the rEBUS probe in lesion localization (Figures 1D–F). After the lesion was located, the rEBUS probe was withdrawn, the guide sheath was reserved, and the forceps were inserted into the guide sheath to reach the lesion. The site of the forceps was reconfirmed by fused fluoroscopy. Five to ten samples were collected from each site. The patients were closely monitored for hemorrhage during operation. An occlusive balloon was prepared for possible massive bleeding prevention. For lesions located in sites that could not be reached by rEBUS, a 2.8 mm bronchoscope was used to reach the lesions under the guidance of fused fluoroscopy. After the biopsy, the samples were submitted for etiologic testing and were fixed in formalin solution for pathological examination. Chest radiography was performed 4 h postoperatively to exclude pneumothorax.

### Statistical analysis

A descriptive analysis was performed. Enumeration data were presented as number of cases, while measurement data were presented as the range.

## Results

### General data and clinical characteristics

The clinical data of the five children are shown in Table 1. Patient 1 was admitted to hospital due to episodic back pain and presence of peripheral pulmonary Lesions (diameter 12 mm) in the branch of the lateral segment of the right middle lobe on chest CT for more than 2 months (bronchoscopic procedure duration 75 min). Patient 2 had been diagnosed with cervical rhabdomyosarcoma and experienced a dry cough without pyrexia during chemotherapy. Chest CT of Patient 2 revealed a solid nodule (3 × 2.7 cm) in the branch of the lateral basal segment of the left lower lobe (Figure 1B) (bronchoscopic procedure duration 55 min). Patient 3 was admitted to hospital due to the discovery of a ground-glass opacity peripheral Pulmonary Lesions (diameter 9 mm) in the dorsal segment, medial sub-segment, of the right lower lobe 3 weeks prior (bronchoscopic procedure duration 70 min). Patient 4 was admitted to hospital because of a cough for more than 40 days. Chest CT of Patient 4 showed enlargement of the left pulmonary hilar and mediastinal lymph nodes and scattered solid nodules in both lungs (bronchoscopic procedure duration 45 min). Patient 5 was admitted to hospital with recurrent pyrexia for more than 10 days after undergoing allogeneic bone marrow transplantation 5 months ago. CT of Patient 5 showed multiple ground-glass opacity nodules; biopsy samples were collected from the lateral basal segment of the left lower lobe (bronchoscopic procedure duration 50 min).

**Table 1:** Clinical data of children who underwent archimedes virtual bronchoscopic navigation-guided lung biopsy.

Case No.	Gender	Age (years)	Biopsy sites	Pathology/pathogen results	Guidance	Complications
1	Male	9.1	Right middle lobe lateral sub-segment	Epstein-Barr virus infection related lymphoproliferative disease	Archimedes, REBUS	No
2	Male	15.8	Left lower lobe lateral basal sub-segment	Pulmonary metastasis of rhabdomyosarcoma	Archimedes, REBUS	No
3	Male	9.6	Right lower lobe dorsal segment medial sub-segmental sub-branch	Pulmonary vasculitis	Archimedes, REBUS	No
4	Male	7.1	Right lower lobe posterior sub-segment	Inflammatory granuloma /Mycobacterium tuberculosis	Archimedes, REBUS	No
5	Male	8.2	Left lower lobe lateral basal sub-segment	Inflammatory granuloma /Aspergillus	Archimedes, REBUS	No

REBUS, radial endobronchial ultrasound.

### Biopsy results

**Figure 2:**
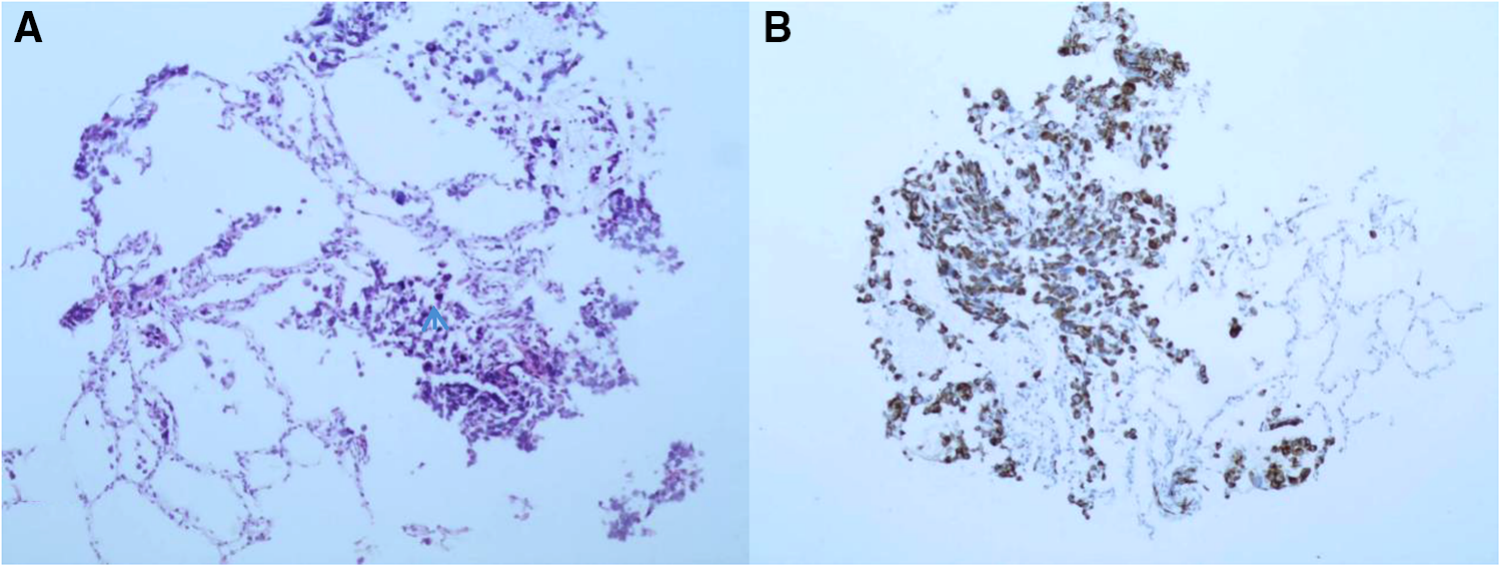
Histopathological examination of biopsy specimens in patient 2 showing rhabdomyosarcoma lung metastasis. (**A**) hematoxylin and eosin staining, ×100; (**B**) immunohistochemistry staining, ×100.

Biopsy samples were successfully obtained from all five children. The pathological results showed Epstein-Barr virus infection-related lymphoproliferative disease in Patient 1, pulmonary metastasis of rhabdomyosarcoma (Figures 2A,B) in Patient 2, and pulmonary vasculitis in Patient 3. For Patient 4, pathological examination of the biopsy tissue sample revealed inflammatory granuloma, and etiologic high-throughput sequencing showed *Mycobacterium tuberculosis*. For Patient 5, pathological examination suggested inflammatory granuloma, and etiologic high-throughput sequencing indicated *Aspergillus*.

### Complications

None of the five children experienced complications, including hemoptysis and pneumothorax.

## Discussion

With the advent and development of bronchoscopic navigation, the localization accuracy and diagnostic rate of transbronchial lung biopsy have significantly increased (10). A controlled study of the biopsy of 10 pulmonary peripheral lesions demonstrated that the accuracy was 94% ± 7.9% for VBN-guided localization compared with 43% ± 16% for conventional bronchoscopy (11). Furthermore, the mean biopsy site positional error (distance from the forceps contact point to the ground truth lesion boundary) was 2.2 ± 2.3 mm for VBN-guided localization and 9.7 ± 9.1 mm for conventional bronchoscopy (12). Archimedes is a virtual bronchoscopic technique that reconstructs CT images to display a 3D bronchial tree, and automatically calculates guide paths to lesions. This navigation system provides information regarding the presence of vessels outside the airway walls, bronchial diameter, bronchoscopic path distance, lesion size, and distance from the lesion to the nearest pleura in the virtual bronchoscopic view. Archimedes could synchronously display the virtual bronchoscopic images and the actual video, and enable an electronic guide path to be overlaid onto the endoscopic image. In addition, the fused fluoroscopy technique can integrate real-time fluoroscopy data with the 3D CT data during the procedure, achieving real-time intraoperative guidance for the operator. This navigation system significantly increases the diagnostic accuracy of bronchoscopy for peripheral pulmonary lesions and enables the minimally invasive diagnosis of lung nodules. In comparison, rEBUS can accurately localize pulmonary peripheral lesions, but can only confirm the lesions rather than guiding the localization; therefore, the combination of rEBUS with VBN further increases the diagnostic rate and safety (9, 10). Archimedes has been widely applied in diagnostic lung biopsy in adults (10, 12), but its application in children has not been reported.

The present results demonstrated that Archimedes was feasible in assisting safe and accurate lung biopsy procedures in children. The following suggestions are based on our experience obtained through this study. First of all, Archimedes system is an auxiliary system of bronchoscopy, which helps guide bronchoscope to reach the target lesion rapidly during operation. At the same time, the system can also confirm the relative position of the target lesion and bronchoscope in real time by using fluoroscopy through fused imaging technology. The navigation path automatically generated by the Archimedes system could check and adjust by the operator if necessary. We drew the navigation path map manually according to the CT image, and compared and adjusted it with the navigation path automatically generated by Archimedes system, to further improve the success rate of the operation. Second, in clinical practice, considering the possible errors in the planning path of the navigation system, the operator should use rEBUS to confirm the target lesion after reaching the target area. For the diagnosis of peripheral pulmonary lesions, we usually used an Olympus P290 bronchoscope. The working part of the bronchoscope has an outer diameter of 4.0 mm, and the diameter of its working channel is 2.8 mm. The rEBUS probe and guide sheath are allowed to be used. When arrived at the target lesion according to the guidance of Archimedes system, we placed the guide sheath and rEBUS probe along the working channel of bronchoscope, and confirmed the target lesion through rEBUS. At the same time, we also used the fused fluoroscopy of Archimedes system to confirm the target lesion location again by using C-arm x-ray positioning. For the ground glass density lesions, the acoustic images of rEBUS sometimes lack recognizable features, in which case x-ray imaging is essential. When the operator confirmed that he had reached the target lesion, he withdrew the rEBUS probe and left the guiding sheath *in situ*. The operator then placed biopsy forceps or cell brushes through the guide sheath to complete the sample collection. Sometimes, the bronchus where the target lesion is located is too small, and it is difficult for a bronchoscopic probe with an outer diameter of 4 mm to enter. In this case, a bronchoscope with an outer diameter of 2.8 mm can be used instead. However, since the working channel of this ultrathin bronchoscope is only 1.2 mm, the rEBUS probe can no longer be used, and the relative position between the bronchoscope and the lesion can only be determined by fused imaging technology and x-ray multiaxial fluoroscopy. In this case, cone beam CT may be a better imaging aid. It is worth noting that the sample size taken by conventional biopsy forceps is small. For some difficult and complicated cases, it is difficult to obtain an effective diagnosis if the sample size is too small. Therefore, how to obtain larger and higher quality biopsy specimens under bronchoscopy has been another challenge. Recently, some scholars have explored the application of transbronchial cryo lung biopsy in the field of pediatrics (4). The cryo biopsy showed better diagnostic efficacy and good safety. In the future, the combined application of a bronchoscopic navigation system and cryo biopsy technology is worth exploring and may become a more effective and safe diagnostic method. Third, during the operation, there are some factors may affect the effectiveness of the navigation system. For example, changes in respiratory movement and lung volume may cause displacement of the lesion location determined by the navigation system, especially for lesions located in the bilateral inferior lobe. The respiratory gating technique matched with the navigation system has not been applied yet. To reduce the impact of respiratory movement and changes in lung volume on the efficiency of the navigation system, it is important to maintain stable ventilation during operation. Therefore, tracheal intubation may be a more appropriate ventilation support method for general anesthesia. Tracheal intubation may limit the pulmonologist's operation of the bronchoscope, so a laryngeal mask is an alternative ventilation support method. The use of a laryngeal mask requires the pulmonologist and anesthesiologist to have more experience. In addition, excessive airway secretions may also affect the efficiency of the navigation system. In this case, the distal bronchial lumen may be blocked or even occluded by secretions, which makes it difficult for the operator to find the terminal bronchus where the target lesion is located according to the guidance of the navigation system. Proper clearance of airway secretions and clear vision under the bronchoscope are very important for a successful operation. Sometimes the method of blowing air through the bronchoscope working channel can be used to keep the distal bronchioles open, to facilitate the rEBUS probe or guide sheath to enter the target bronchus accurately. Fourth, in the field of pediatrics, the safety of interventional diagnostic technology is the focus of clinical attention. The common complications of transbronchial lung biopsy are hemorrhage and pneumothorax. Archimedes system can reconstruct the bronchial tree and accompanying vascular tree in three dimensions, which is helpful for the operator to more accurately understand the blood supply around the target lesion, so as to select a safer biopsy location or method (forceps biopsy or needle aspiration biopsy). In addition, the Archimedes system can also calculate the distance from the target lesion to the pleura, so as to help the operator determine the appropriate depth of biopsy forceps and reduce the risk of pneumothorax. The use of guiding sheath during operation can further improve the safety and success rate of operation. There were no complications of hemorrhage and pneumothorax in the 5 patients in our study.

In conclusion, in these 5 patients, Archimedes guided bronchoscopic lung biopsy was a feasible and efficient way in children. However, as the sample size was small, the findings require verification in clinical studies with larger sample sizes. We believe that with future technical development, Archimedes-guided lung biopsy will become more important and extensively used in bronchoscopy for children.

## Data Availability

The original contributions presented in the study are included in the article/Supplementary Material, further inquiries can be directed to the corresponding authors.
